# Application of Genomics to Prevent and Combat Emerging Infectious Disease Threats in Latin America: Perspectives from an Overseas U.S. Navy Research Command

**DOI:** 10.3390/tropicalmed11090245

**Published:** 2026-08-28

**Authors:** Hugo O. Valdivia, Maria Silva, Cristopher D. Cruz, Paul Rios, Marisa E. Lozano, Julia S. Ampuero, Yeny Tinoco, Jeffrey Spiro, Yuliya S. Johnson, Alden S. Estep, Steev Loyola, Carmen Flores-Mendoza, Gissella M. Vasquez, Jose A. Garcia-Rivera, Henju Marjuki

**Affiliations:** 1 U.S. Naval Medical Research Unit SOUTH, Lima 07006, Peru; hugo.o.valdivia.ln@health.mil (H.O.V.); paul.a.rios12.ln@health.mil (P.R.); julia.s.ampuero.ln@health.mil (J.S.A.); steev.o.loyola.ln@health.mil (S.L.);; 2 Culmen Inc., Alexandria, VA 22314, USA; 3 Center for Medical, Agricultural and Veterinary Entomology, United States Department of Agriculture’s Agricultural Research Service, Gainesville, FL 32608, USA; alden.estep@usda.gov

**Keywords:** genomic surveillance, emerging infectious diseases, high-throughput sequencing, vector-borne diseases

## Abstract

Endemic and (re)emerging infectious diseases pose a major threat to public health and military readiness in Central and South America. The high biodiversity and the differing transmission pathways require the use of highly sensitive methods to track the emergence and spread of these diseases. High-throughput sequencing technologies have become a critical resource for the field of infectious disease monitoring, specifically for tracking pathogen evolution, antimicrobial resistance, and transmission. This review provides an overview of the implementation of genomics-informed infectious disease surveillance conducted by the U.S. Naval Medical Research Unit SOUTH, focusing on operational, infrastructural, and logistical challenges in resource-limited settings and outlining strategies for building sustainable regional capacity. Selected case studies focused on prominent biothreats including dengue, Oropouche, malaria, and antimicrobial resistance demonstrate the significance and applicability of targeted and agnostic sequencing approaches to elucidate transmission pathways, pathogen discovery, and guide public health responses and field-deployable applications.

## 1. Introduction

Endemic and (re)emerging infectious diseases pose significant challenges to global health and biosecurity. Modern demographic and ecological conditions, such as population growth, globalization, urbanization, and increased interactions between humans and animals, facilitate the spread of these diseases [1]. Integrating pathogen genomics with epidemiology transforms disease surveillance from reactive measures to proactive, high-resolution pathogen intelligence. This approach provides actionable insights, enabling public health agencies to track emerging threats with exceptional precision [2].

Genomic surveillance utilizes next-generation sequencing (NGS) and is part of a broader system for disease control and prevention, reinforcing end-to-end capabilities including sample collection, diagnostics, data sharing, and analysis [3]. Recent advances in sequencing technologies and bioinformatics have made high-resolution pathogen tracking and comparative genomic analysis possible. This approach allows for continuous monitoring of genetic changes in pathogens, which is vital for tracking their evolution, spread, and public health impact. An essential component of genomic surveillance is the identification of emerging genetic variants, such as those observed in SARS-CoV-2. This information is crucial for guiding vaccine development and treatment strategies by analyzing how mutations affect virulence and the potential for person-to-person transmission [3,4]. NGS can be applied directly to clinical or environmental samples, offering high confidence in identifying individual base changes, even in cases of mixed infections. Bioinformatics tools analyze sequence data and establish important linkages between pathogens, animals, humans, and geographic locations [2].

Latin America (South America, Central America, Mexico, and parts of the Caribbean) faces a significant burden of infectious diseases, largely driven by vector-borne pathogens such as dengue, Zika, chikungunya, and Oropouche viruses, as well as neglected tropical diseases and sexually transmitted infections [5]. The implementation of NGS approaches is vital for countries in the region, enabling precise, real-time tracking of transmission dynamics. This enhances public health responses to vector-borne and other infectious diseases, especially in a region significantly affected by climate-related outbreaks [2,6]. To this end, international collaborative efforts are essential to successfully develop genomic surveillance systems in the region, creating the political will needed to make this sophisticated tool accessible to low- and middle-income countries [7]. Currently, several Latin American countries, such as Brazil, Mexico, and Chile, and to some extent, Colombia, Peru, and Argentina, possess relatively robust high-throughput sequencing capabilities that are well integrated into national public health initiatives. Although genomic capabilities have grown, there is a recognized need to translate data into public health policies, strengthen bioinformatics expertise, and promote equitable partnerships where Latin American institutions participate as equal partners in research [8].

The U.S. Naval Medical Research Unit SOUTH (NAMRU SOUTH), formerly known as NAMRU-6, is the Department of War’s premier medical research command in Latin America and the only U.S. military medical research command dedicated to supporting the U.S. Southern Command (SOUTHCOM) Area of Responsibility, encompassing Central America, South America, and the Caribbean [9]. Headquartered in Lima, Peru, NAMRU SOUTH is committed to protecting the health and operational readiness of U.S. forces while strengthening regional health security through biomedical research, infectious disease surveillance, outbreak response, medical countermeasure evaluation, and global health engagement.

Established in Peru in 1983, NAMRU SOUTH has built more than four decades of scientific excellence and enduring partnerships throughout the region [9]. The command works closely with ministries of health, military medical services, academic institutions, and international organizations to address infectious disease threats that impact both military readiness and public health. Through these collaborations, NAMRU SOUTH has earned a reputation as a trusted and reliable partner, advancing scientific knowledge while enhancing regional capacity to detect, prevent, and respond to emerging health threats.

**Figure 1:**
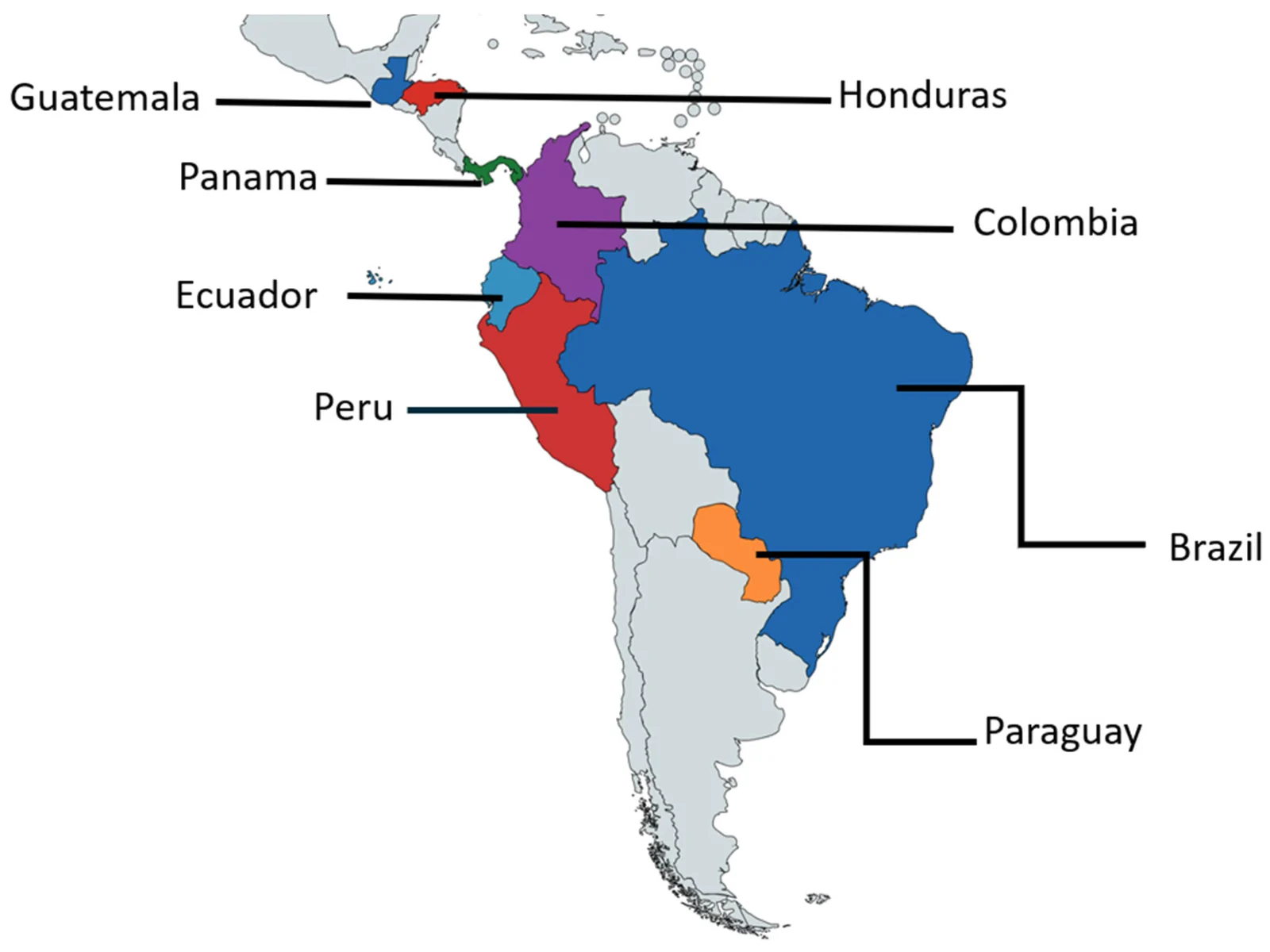
U.S. Naval Research Medical Unit (NAMRU) SOUTH collaborative network in South and Central America between 2024–2025. Colors denote countries where NAMRU SOUTH conducts collaborative studies.

NAMRU SOUTH’s multidisciplinary teams conduct operationally relevant research on a broad range of infectious diseases, including vector-borne, respiratory, enteric, and febrile illnesses that pose risks to deployed forces and vulnerable populations. The command maintains extensive surveillance networks across the region that provide critical early warning capabilities for emerging and re-emerging infectious diseases (Figure 1). These networks enable rapid detection, characterization, and response to outbreaks, generating actionable data that informs military force health protection decisions and public health interventions. By delivering high-quality research, strengthening partner nation capabilities, and providing timely surveillance of infectious disease threats, the command remains at the forefront of efforts to enhance regional health security and ensure the readiness of U.S. and partner nation forces throughout the Western Hemisphere.

## 2. NAMRU South’s History, Experience and Challenges with NGS

**Figure 2:**
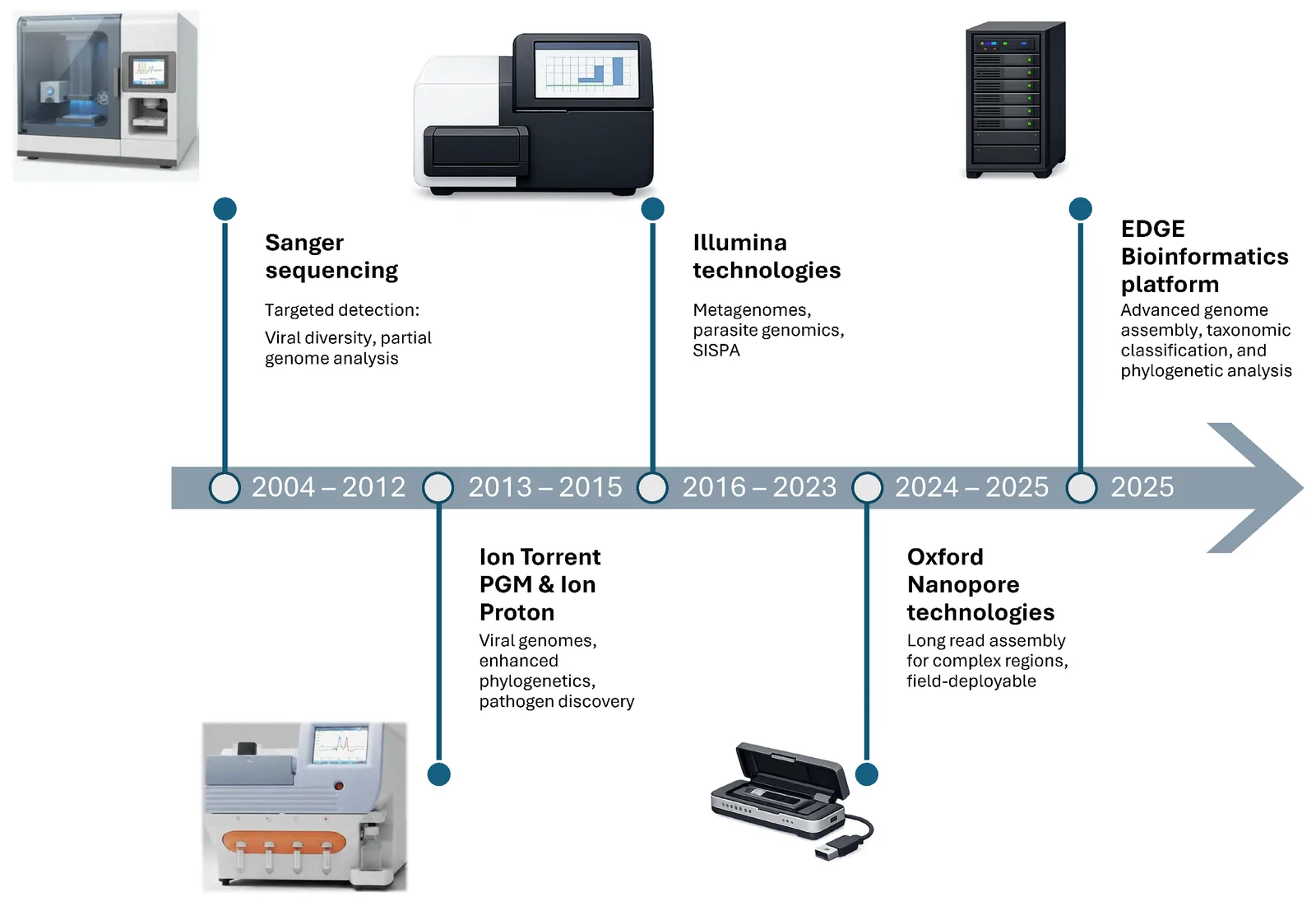
Deployment of sequencing capabilities at NAMRU SOUTH (2004–Present) mapping the transition from low-throughput to high-throughput genomic surveillance and field-deployable platforms. Image created using Gemini Enterprise implemented in GenAI (July 2026).

Over the last decade, NAMRU SOUTH has undergone a major technology transition from low-throughput sequencing methods to NGS technologies (Figure 2), changing the paradigm for biomedical research and infectious disease surveillance from targeted detection to unbiased pathogen discovery and full genomic characterization of circulating pathogens.

Prior to the adoption of NGS technologies, molecular surveillance of febrile and emerging infections relied primarily on genetic amplification-based (i.e., polymerase chain reaction [PCR]) technologies and Sanger-based sequencing approaches. Beginning in 2004, these efforts supported the genetic characterization of pathogens of regional public health importance, including dengue viruses, influenza viruses, alphaviruses (e.g., Venezuelan equine encephalitis and Mayaro viruses), and orthobunyaviruses (e.g., Oropouche, Guaroa, and Group C viruses). During this period, molecular and gene-targeted sequencing methods contributed to the identification of emerging pathogens, characterization of viral diversity, and investigation of outbreaks throughout Peru and neighboring countries. Studies describing the molecular epidemiology of dengue virus lineages in South America [10,11], investigations of rickettsial pathogens [12,13], and the identification of novel arboviruses circulating in the Peruvian Amazon [14,15] helped establish expertise in molecular surveillance, phylogenetics, and pathogen discovery that later facilitated implementation of NGS-based approaches.

Although NGS offers clear advantages for infectious disease research and surveillance, establishing and maintaining these capabilities in South America presents unique operational, infrastructural, and logistical challenges. The increasing use of high-throughput technologies results in massive data sets that often surpass local capacities for data storage and analysis, requiring complex computing resources and highly skilled bioinformaticians, which are scarce in the region due to high turnover, difficulty retaining specialized personnel, and a limited number of specialized training opportunities. To mitigate the data analysis limitation, research institutions such as NAMRU SOUTH are implementing “train-the-trainer” initiatives that address both wet-lab and downstream bioinformatics analysis. This approach builds local and sustained capacity and lays the groundwork for training new personnel [16]. This strategy can be further supplemented with online resources, workshops and active participation in regional conferences and networks (such as those organized by the Iberoamerican Society for Bioinformatics [SoiBio], the International Society for Computational Biology [ISCB] Latin America or the Brazilian Association for Bioinformatics and Computational Biology [AB3C]) to foster long-term retention and collaboration.

Another component of the analytical bottleneck is the mismatch between the growth in NGS data and the limited hardware infrastructure available in many regional laboratories in terms of computational power, data storage, and network bandwidth. To actively address this challenge, NAMRU SOUTH has recently established a Sequencing Core Facility to provide a cost-efficient computing architecture that centralizes high-performance local servers coupled with Edge computing for rapid on-site analysis of genomic data. This system allows data to be processed and stored closer to where it is generated. Other institutions can potentially adopt a hybrid model combining cost-effective cloud computing for intensive primary processing (such as raw data processing, assembly or large-scale projects) paired with local high-performance laptops for secondary data analysis.

Many local institutions in the region face additional challenges regarding the logistics of the supply chain. Most NGS platforms, specialized kits, and reagents must be imported from the United States and Europe through a limited number of local distributors. This results in extended delivery timelines, potential cold chain problems, and an increase in costs due to customs and importation processes and fees. To address these bottlenecks, long-term bilateral collaborations across the US and Europe with local institutions in South and Central America can streamline regional logistics and build a more resilient distribution network that can significantly reduce operational costs.

## 3. Building a Regional Genomics Hub at NAMRU SOUTH

The successful implementation of a sustainable sequencing core depends on the integration of laboratory infrastructure, trained personnel, bioinformatics resources, and long-term institutional commitment. At NAMRU SOUTH, the development of NGS capabilities was built upon more than a decade of experience in molecular epidemiology, pathogen surveillance, and viral discovery (Figure 2).

As genomic technologies became increasingly important for infectious disease surveillance, NAMRU SOUTH initiated a gradual transition toward WGS. This evolution began in 2013 with the acquisition of the Ion Torrent Personal Genome Machine and Ion Proton platforms, enabling routine generation of complete viral genomes and expanding surveillance beyond the partial genomic regions traditionally analyzed using Sanger sequencing.

The sequencing portfolio expanded with the incorporation of Illumina technologies between 2016 and 2018. Compared with earlier sequencing systems, these platforms introduced paired-end sequencing capabilities that improved genome assembly, increased sequencing accuracy, and expanded the range of surveillance applications that could be supported. More recently, NAMRU SOUTH incorporated a MiSeq Dx instrument and Oxford Nanopore Technologies (ONT) MinION sequencing devices, providing additional flexibility for both routine genomic surveillance and emerging field-based sequencing activities.

As sequencing throughput increased, the need for local bioinformatics capacity became equally important. Processing and interpreting genomic datasets required infrastructure that extended beyond the traditional laboratory bench. To address these needs, NAMRU SOUTH deployed a dedicated bioinformatics server in 2019 and implemented the EDGE Bioinformatics platform in collaboration with the Naval Medical Research Command Biological Defense Research Directorate (BDRD). This partnership also supported training opportunities that strengthened local expertise in genome assembly, taxonomic classification, and phylogenetic analysis.

The core of our current computational resources is a high-performance bioinformatics server running AlmaLinux, equipped with 256 CPU cores, 1 TB of RAM, and 21 TB of dedicated disk storage. This centralized high-performance computing (HPC) unit is further complemented with six Linux-based workstations running various Long-Term Support (LTS) Ubuntu distributions (versions 20.04 to 24.04), providing a combined capacity of 104 CPU cores, 283 GB of RAM, and 13 TB of disk storage. These workstations allow for decentralized, parallel pipeline development, local database queries, and small-to-medium scale analysis.

The implementation of NGS-based pathogen discovery and WGS for surveillance of acute febrile illnesses and respiratory infections has been critical for identifying emerging threats and understanding viral introduction pathways (Table 1). For example, agnostic sequencing enabled NAMRU SOUTH to identify a novel Echarate virus variant in the western Peruvian Amazon isolated from a patient presenting with acute febrile illness characterized by high fever, severe headache, and systemic myalgia [17]. In 2022, NAMRU SOUTH also performed whole-genome sequencing and confirmed the first case of highly pathogenic avian influenza A (H5N1) in Peru in collaboration with local animal health authorities [18]. More recently, in 2023, the use of unbiased NGS allowed NAMRU SOUTH to identify the co-circulation of two Oropouche (OROV) virus lineages during an outbreak in the Loreto region in the Peruvian Amazon [19].

In addition to its major contributions to infectious disease research, NGS has also advanced the field of parasitology by enabling high-resolution characterization of *Plasmodium vivax* and *Plasmodium falciparum* populations. This has shifted surveillance from focusing on individual drug resistance mutations to analyzing whole-genome profiles, allowing for the identification of selective sweeps, determination of geographic origin and introduction events, and monitoring of population structure across evolving transmission settings [16,17,18,19].

## 4. Comparative Analysis of Sequencing Tools at NAMRU SOUTH

The expansion of NGS capabilities has been driven by the diverse research and surveillance objectives of NAMRU SOUTH. Because no single sequencing strategy is optimal for every scenario, NAMRU SOUTH has implemented a multifaceted framework that integrates short-read and long-read technologies, together with targeted amplification and agnostic sequencing approaches. The selection of a particular workflow depends on several factors, including the pathogen of interest, sample quality, turnaround time requirements, and the specific epidemiological or research question being addressed.

As previously noted, Illumina short-read sequencing platforms support most routine genomic surveillance activities conducted at NAMRU SOUTH. The current sequencing infrastructure includes MiSeq, MiSeq Dx, and MiniSeq instruments (with potential acquisition of NextSeq sequencing systems), which generate highly accurate paired-end sequencing data (>99.9% base-calling accuracy), which is essential for whole-genome characterization of viral and bacterial pathogens. These platforms have been routinely used for consensus genome generation, variant detection, phylogenetic analyses, molecular epidemiology studies, and antimicrobial resistance investigations.

Over the past several years, Illumina-based workflows have been applied to surveillance of dengue virus, influenza viruses, SARS-CoV-2, and other pathogens of public health importance, while also supporting bacterial WGS and metagenomic investigations. Their principal strengths include high accuracy, robust analytical pipelines, and compatibility with established public health surveillance frameworks. The main limitations are the need for dedicated laboratory infrastructure, trained personnel, and longer turnaround times compared with portable sequencing technologies. From a bioinformatics perspective, the short read lengths (typically 150–300 bp) generated by these technologies are insufficient to resolve highly repetitive genomic elements or complex structural variations that are highly abundant in *Trypanosoma*, *Leishmania* and *Plasmodium* genomes as well as arthropod vectors.

More recently, ONT MinION devices were incorporated into the sequencing portfolio at NAMRU SOUTH to support emerging surveillance needs that required greater operational flexibility and portability. As the laboratory expanded into activities involving field-based surveillance and outbreak investigations, access to a portable sequencing platform became increasingly important. In addition, the long-read capability of Nanopore sequencing (>10 kb) overcomes the assembly limitations of short reads, providing new opportunities for the characterization of bacterial and parasitic pathogens, where larger and more complex genomes may benefit from longer sequencing reads. Following implementation, targeted sequencing workflows for influenza viruses and SARS-CoV-2, as well as Sequence-Independent, Single Primer Amplification (SISPA) protocols, were evaluated using Nanopore sequencing and compared against established Illumina-based workflows to assess their suitability for routine surveillance and pathogen discovery applications.

Likewise, surveillance of arthropod vectors has greatly advanced with the implementation of ONT sequencing at NAMRU SOUTH, allowing for simultaneous identification of vector species, pathogens, and hosts in a shorter timeframe than conventional targeted molecular methods and at a lower cost than short-read sequencing technologies. For example, identification of *Bartonella* spp. by multi-locus sequence-typing can be challenging in arthropod samples due to host and complex microbiota background; whereas ONT sequencing provides higher taxonomic resolution and accurate identification of *Bartonella* spp. while reducing turnaround time [20].

**Figure 3:**
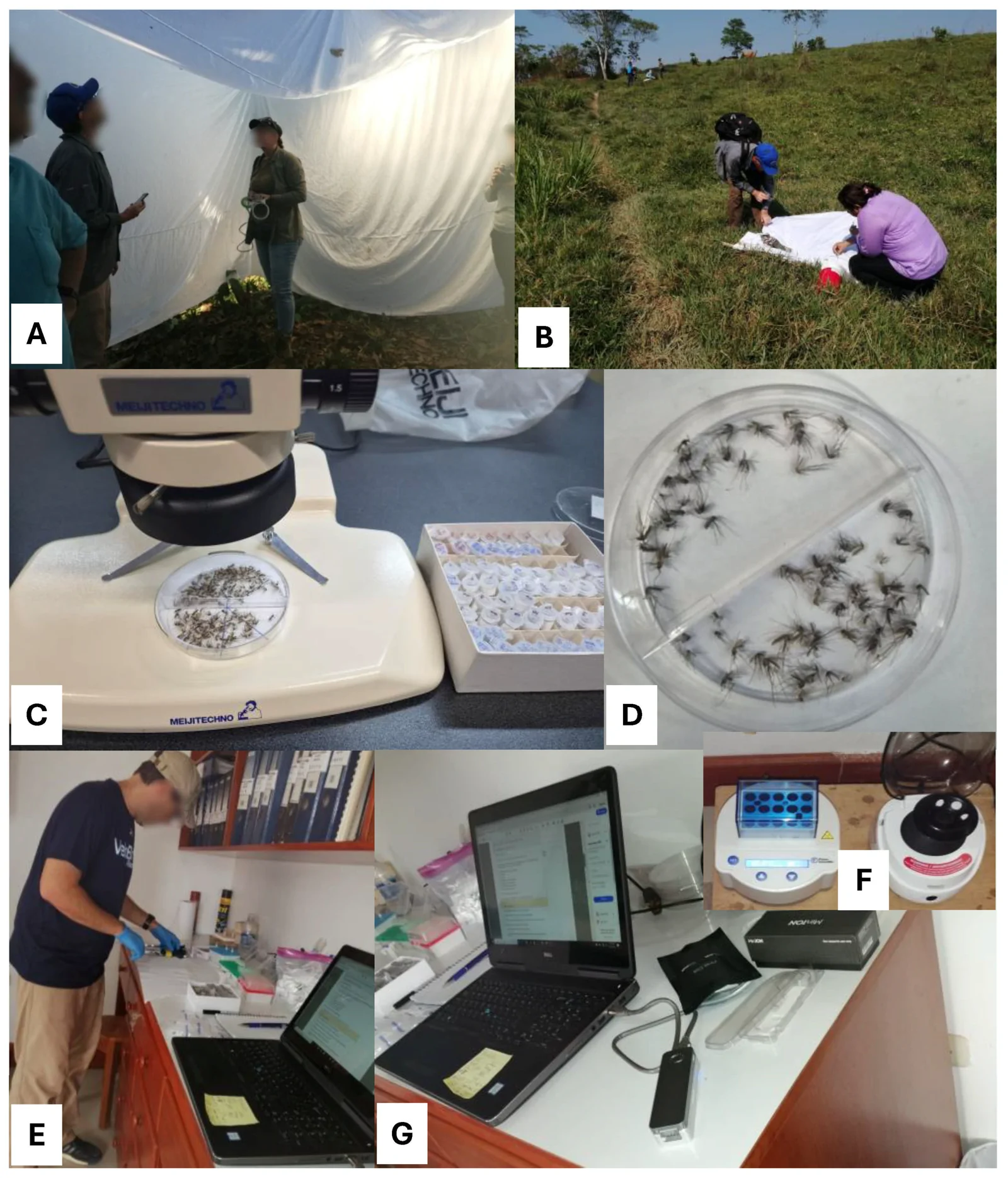
Field-deployable system for mosquito and tick identification using Oxford Nanopore Technologies (ONT) sequencing. (**A**) Mosquito collection using Shannon trap. (**B**) Tick collection by dragging. (**C**) Specimen sorting and morphological identification under a stereoscope. (**D**) Identified mosquitoes for testing. (**E**) Specimen DNA extraction. (**F**) Incubating shaker and minicentrifuge used for DNA extraction and library preparation. (**G**) Portable ONT MinION Mk1B sequencing device.

The portability of MinION devices greatly facilitates identification of vector-borne threats in the field, close to the point of collection, by quickly testing arthropod vectors for pathogens with user-friendly protocols and yielding results in less than 48 h under rugged conditions [21]. This approach is particularly useful during vector-borne disease (VBD) outbreaks given its portability and broad capacity to detect pathogens without a priori knowledge but with high sensitivity, helping to guide healthcare responses. Recently, the operational feasibility of ONT sequencing was tested in the field for mosquito and tick identification in a remote high jungle region in northern Peru (Figure 3), where *Aedes serratus*, *Coquillettidia nigricans*, and *Rhipicephalus microplus* were identified (99.2–100% identity) using BoldSystems [22]. One early limitation was the higher error rate relative to other sequencing technologies; however, improved chemistry and advances in base calling algorithms over the past few years have increased the read accuracy to approximately 99% [23]. Another consideration is that the ability to identify a pathogen in a sample depends on the nucleic acid concentration, which may be limited for pathogens infecting arthropods.

Beyond portability, long-read sequencing can facilitate the assembly of repetitive or structurally complex genomic regions that are difficult to reconstruct using short-read data alone. Although ONT sequencing generally exhibits lower per-read accuracy than Illumina platforms and often requires additional bioinformatic processing to generate high-quality consensus sequences, it provides valuable flexibility for specific surveillance and research applications.

For routine surveillance of known pathogens, targeted amplification has remained one of the most practical and cost-effective sequencing strategies implemented at NAMRU SOUTH. By enriching pathogen-specific nucleic acids before sequencing, these workflows facilitate genome recovery from clinical specimens with low pathogen abundance while reducing the sequencing depth required per sample. Over the past decade, the laboratory has developed and validated targeted whole-genome amplification protocols for dengue virus and Zika virus [24,25], while adapting similar approaches for influenza A and B viruses and SARS-CoV-2. These efforts resulted in over 729 SARS-CoV-2 sequences generated and shared between 2021 and 2026, covering major variants of concern, such as Alpha, Gamma, Delta, and Omicron sequences from Peru, Paraguay, and Colombia, as well as a smaller number from Honduras, Guatemala, and Panama.

The principal limitation of targeted sequencing is that successful genome recovery depends on prior knowledge of the pathogen and the availability of appropriate primer schemes, making these approaches less suitable for investigating unexpected pathogens, highly divergent variants, or mixed infections that may not be efficiently amplified using existing primer sets.

To address this limitation, NAMRU SOUTH implemented SISPA and shotgun metagenomic sequencing workflows. These approaches have supported the characterization of previously unrecognized viral pathogens and variants, including Saffold virus (typically associated with pediatric acute respiratory and gastrointestinal symptoms) [26] and the recently described Echarate virus variant [17]. They have also been incorporated into outbreak investigations where conventional diagnostic testing alone was insufficient to fully characterize the infectious agents involved. One example was the comprehensive microbiological and metagenomic investigation conducted during the Guillain–Barré syndrome outbreak in 2019 in Lima, Peru [27]. While metagenomics successfully detected low-prevalence pathogen co-infections and ruled out suspected viral triggers, the use of WGS conclusively identified clonal strains of *Campylobacter jejuni* (ST2993) as the primary etiological trigger.

Taken together, targeted and agnostic sequencing approaches provide complementary capabilities that support the full spectrum of genomic surveillance activities at NAMRU SOUTH. Targeted workflows remain the preferred strategy for routine monitoring of known pathogens, whereas SISPA and shotgun metagenomic approaches provide critical capacity for pathogen discovery, outbreak investigation, and characterization of emerging infectious disease threats.

## 5. Critical Regional Gaps to Advance NGS for Disease Detection and Prevention

NGS has the potential to significantly augment the field of infectious disease research. However, major systemic gaps need to be addressed to fully harness this potential. The most important critical need is the establishment of robust and standardized NGS wet-lab and bioinformatic pipelines for agnostic sequencing that can be readily implemented across the region. Current targeted molecular diagnostic panels are restricted by their pre-defined primer and probe designs, rendering them blind to variants with mutations on target sequences and novel pathogens. Consequently, the development and integration of a standardized, unbiased metagenomic approach can complement existing panels with simultaneous detection of known, mutated, and entirely novel infectious agents as new threats emerge.

Another major gap, especially in the field of febrile and vector-borne disease surveillance, is that data collection is skewed towards urban centers due to the lack of infrastructure and trained personnel in remote settings and rural communities like in the Amazon Basin. This results in an overrepresentation of pathogen data from large cities instead of high-risk austere settings. For instance, the five arenaviruses that infect humans in Central and South America are predominant in rural and remote areas, such as the areas where NAMRU SOUTH first identified the Chapare arenavirus [28]. Similarly, isolated cases of Ilheus virus [29] and leishmaniasis outbreaks among Peruvian army cadets [30] were reported from remote communities, underscoring the hidden risk in these settings. These remote locations serve as primary corridors for malaria and emerging arboviruses such as OROV and Mayaro and are areas of rural transmission cycles of dengue and yellow fever [19,31]. Capturing real-time NGS data from these remote locations requires the use of innovative cost-effective approaches such as transitioning to field-deployable and portable NGS platforms coupled with cloud-based computing for data analysis.

In bacteriology, NGS has been utilized for various research and clinical applications, but in highly polymicrobial specimens, such as stool, significant challenges remain, including the standardization of sample processing, transportation, and genomic DNA extraction. Furthermore, in data analysis, discriminating between commensal colonizers and actual pathogens necessitates the use of established pipelines to characterize functional profiles and unclassified bacterial genes. Since many microbial genes and taxa remain unclassified, utilizing expanded databases or advanced sequencing methodologies is essential [32].

For arthropod vectors, the use of ONT NGS could be far-reaching through standardized bioinformatics pipelines optimized to detect low-abundance pathogens within samples dominated by a mix of host and environmental DNA. ONT has developed a user-friendly application (Epi2Me) for data analysis; however, preliminary analyses performed at NAMRU SOUTH indicate that metagenomic classifications require confirmation using complementary analytical approaches. To overcome this limitation, pipelines that integrate multiple validation steps were implemented to improve specificity while maintaining rapid turnaround times. Another critical need is the expansion of curated reference databases containing high-quality genomes from medically important vectors and vector-borne pathogens circulating in the region. More comprehensive databases would improve taxonomic assignment, reduce ambiguous classifications, and facilitate discrimination among closely related pathogens. Sample processing capacity is another challenge for vector and vector-borne pathogen surveillance when using the MinION device for metagenomics in the field.

Finally, there is an urgent need to integrate and establish uniform wet-lab protocols and bioinformatic pipelines to allow regional data comparison. For instance, different NGS-based approaches have been developed for malaria surveillance, which are currently in use to collect data by different groups across Central and South America. Although these methods have substantially contributed to our understanding of malaria transmission, these protocols cannot be easily transferred as they were designed for different transmission scenarios and capabilities. Furthermore, data from these studies cannot be easily integrated because of differences in sequenced regions, preventing integrated comparative approaches. Recently, a regional Malaria Molecular Surveillance network (MMS Americas) was established to promote sharing of expertise, integration of protocols, and bioinformatic pipelines, ensuring that sequence data from diverse partners can be reliably aggregated and analyzed [33].

## 6. Use of NGS in Pathogen Detection, Characterization, and Disease Outbreak Investigation

### 6.1. Case Study 1: Genomic Epidemiology of Dengue and Oropouche Viruses in Peru

NAMRU SOUTH long-standing febrile surveillance across Central and South America demonstrated that dengue outbreaks were linked to repeated viral introductions rather than isolated local transmission events, with studies of DENV-3 and DENV-4 showing movement of viral lineages throughout northern South America and highlighting close epidemiological connections between Peru and neighboring countries [11,34]. Subsequent work on the genotype III (American–Asian) of DENV-2 showed the establishment and expansion of two major lineages associated with separate introduction events and epidemic activities in Peru [10]. These studies established an important framework for understanding how dengue viruses are introduced, maintained, and dispersed across the region.

The transition from partial-gene sequencing to whole-genome approaches provided a more detailed view of these processes. To support this effort, targeted methods were developed to recover complete dengue virus genomes directly from clinical specimens [24]. The availability of whole-genome data improved the resolution of evolutionary analyses and enabled a clearer distinction between locally persisting viruses and newly introduced strains. As additional genomic data accumulated, it became possible to examine viral movement at both regional and national scales with greater confidence.

Even before the implementation of routine NGS workflows, phylogenetic analyses provided important insights into dengue virus circulation in Peru. Studies of DENV-3 genotype III and DENV-4 genotype II, a distinct lineage different from strains previously detected in Peru, demonstrated close genetic relationships between Peruvian isolates and viruses circulating in northern South America, supporting the hypothesis that these lineages were introduced through the northern region of the country as part of broader regional transmission networks [11,34]. Similar findings were later reported for the genotype III of DENV-2, where phylogenetic and molecular epidemiological analyses identified multiple introduction events originating from northern South America as well as the Brazil–Bolivia corridor, followed by the progressive replacement of one viral lineage by another [10].

Furthermore, phylogenetic analysis of DENV-2 and DENV-3 genomes generated through surveillance activities revealed a complex pattern of introductions and onward transmission. Multiple independent introductions of DENV-2 were identified from neighboring countries, including Ecuador, Colombia, Brazil, Bolivia, and Paraguay. Similar analyses of DENV-3 identified several genotype III sublineages circulating in Peru, including lineages associated with introductions from Ecuador and Colombia. In addition, continuous sequencing efforts facilitated the identification of the DENV-2 Cosmopolitan genotype (Genotype II) along the Peru–Brazil border in 2019. Subsequent efforts showed that this lineage rapidly expanded and displaced genotype III throughout the country within a few years.

The same analytical approaches have recently been applied to OROV, an emerging arbovirus that has attracted increasing attention following outbreaks reported in several Central and South American countries. Although OROV has been recognized in Peru for decades, molecular investigations conducted through collaborative surveillance efforts have documented its circulation beyond Peru and provided early evidence of regional connectivity. For example, characterization of an OROV infection detected in Colombia in 2017 demonstrated close phylogenetic relationships with strains previously identified in Ecuador and Peru, suggesting movement of viral lineages across national borders and highlighting the importance of coordinated regional surveillance [35]. More recently, WGS and phylogenetic analyses conducted through NAMRU SOUTH surveillance activities demonstrated the co-circulation of multiple lineages and provided evidence of cross-border movement of OROV into Peru [19], as well as the displacement, within a few months, of lineages previously circulating in the northeastern part of the Peruvian Amazon by the lineage entering at the border between Brazil and Peru (unpublished data).

These findings demonstrated that DENV and OROV outbreaks and circulation in Peru are primarily shaped by continuous regional connectivity rather than long-term local persistence of a single viral lineage, but also the importance of a long-term, sustainable genome surveillance program and analysis in the understanding of the dynamic transmission of viral diseases. Tracking the rapid displacement of one lineage by another, such as the expansion of the DENV-2 Cosmopolitan genotype, serves as an early warning system, allowing the allocation of resources, updating diagnostic testing protocols to prevent false negatives, and tailoring interventions to mitigate the impact of impending epidemics.

### 6.2. Case Study 2: Nanopore Metagenomic DNA Sequencing in Ectoparasites

In 2025, NAMRU SOUTH implemented an ONT-based metagenome sequencing workflow [36] to screen banked ectoparasite vectors (ticks and fleas) collected from dogs across transmission hotspots in Peru between 2023 and 2024 (Table 2). Samples were selected given their high relative abundance in field collections and their role in transmission of *Rickettsia* spp., *Ehrlichia* spp., *Babesia* spp., and tick-borne viruses [37,38]. Following nucleic acid extraction, five of the pools tested positive for *Rickettsia* spp. via quantitative PCR [39] (Table 2).

*Rickettsia*-positive and negative pools were subjected to ONT metagenomic DNA sequencing following the REDI-NET tick-testing protocols on a MinION Mk1C [40]. Initial taxonomic profiling using kraken2 and Bracken matched several reads in a tick pool from Loreto with *Orientia tsutsugamushi*. However, failure to map these reads by Minimap2 with the reference genome (Table 2) suggested a spurious classification rather than true detection. Preliminary metagenomic analyses detected *Rickettsia asembonensis* in two flea pools. Subsequent mapping using species-associated genomic regions (*gltA*, *ompA*, *ompB*, *sca4*) detected reads from the *Rickettsia felis*-like group, suggesting that additional bioinformatic analyses and expanded reference datasets are needed for definitive species assignment. An additional benefit of the workflow was the rapid simultaneous confirmation of vector identity in Minimap2. COI-based mapping confirmed all *Rh. sanguineus* pools and the Amazonas *Ct. felis* pool with 95–100% sequence identity by using a reference (BOLD:AAU2924 and BOLD:AAY6332, respectively). Notably, the Loreto flea pool morphologically identified as *Ct. canis* showed 96–97% identity with *Ct. felis* (BOLD:AAY6332), highlighting the utility of sequencing for resolving taxonomic ambiguities arising from morphological identification.

Overall, these preliminary metagenomic analyses of ticks and fleas, even in a sample with low DNA concentration and high qPCR Ct values, demonstrated the potential of ONT sequencing to simultaneously characterize both arthropod vectors and associated bacterial pathogens within approximately 48 h. Although fully confirmatory bioinformatic analyses are ongoing, these early results illustrate the value of rapid metagenomic screening for prioritizing samples and generating actionable information for surveillance efforts, while providing early situational awareness during vector-borne disease surveillance. Our non-targeted workflow proved operational utility for detecting DNA compatible with members of the *Rickettsia felis*-like group in fleas from Loreto and Amazonas. However, this was not the case for tick samples for which *Rickettsia* species were not identified, although qPCR Ct values indicate otherwise. This may suggest that host backgrounds may reduce sensitivity by diluting pathogen DNA [41]. Also, relying exclusively on EPI2ME and Minimap limits detection of ectoparasite-borne DNA pathogens or can provide incorrect results, as in the preliminary detection of *O*. *tsutsugamushi* in ticks.

Thus, using standardized pipelines and enhanced databases as those from the REDI-NET can overcome this limitation [42]. Rather than representing limitations unique to ONT sequencing, our findings emphasize that taxonomic assignments generated by automated classifiers should be interpreted as preliminary until supported by advanced and independent analytical approaches. Yet, given the limited DNA quantity obtained from arthropod samples, using ONT metagenomic DNA sequencing for simultaneous identification of pathogens and vector species offers a significant technical advantage over conventional single or multiplex PCR approaches that are more time-consuming.

### 6.3. Case Study 3: Tracking Malaria in Diverse Epidemiological Settings

NGS offers powerful tools to understand the dynamics of malaria transmission, moving beyond traditional drug resistance marker identification to population genomics and evolutionary analysis, which are critical in complex and widely diverse epidemiological settings in the Americas. NAMRU SOUTH and its partners have been at the forefront of this effort in the Amazon Basin, supporting the understanding of *P. vivax* and *P. falciparum* in the region.

Key hurdles in malaria genomics, especially for *P. vivax*, are the abundance of “contaminating” human DNA, the low parasite density in clinical samples and the difficulty of in vitro cultivation [43]. To overcome these challenges, NAMRU SOUTH collaborated with the University of California in San Diego on the development and implementation of a Selective Whole-Genome Amplification (SWGA) technique that enriches *P. vivax* DNA directly from unprocessed clinical blood samples and dried blood spots, bypassing the need for leukocyte filtration or bead-based enrichment [44,45]. This method allowed for cost-effective and scalable WGS of *P. vivax* from field and remote environments in the Peruvian Amazon, allowing the capture of up to 95% of the core *P. vivax* genome.

**Figure 4:**
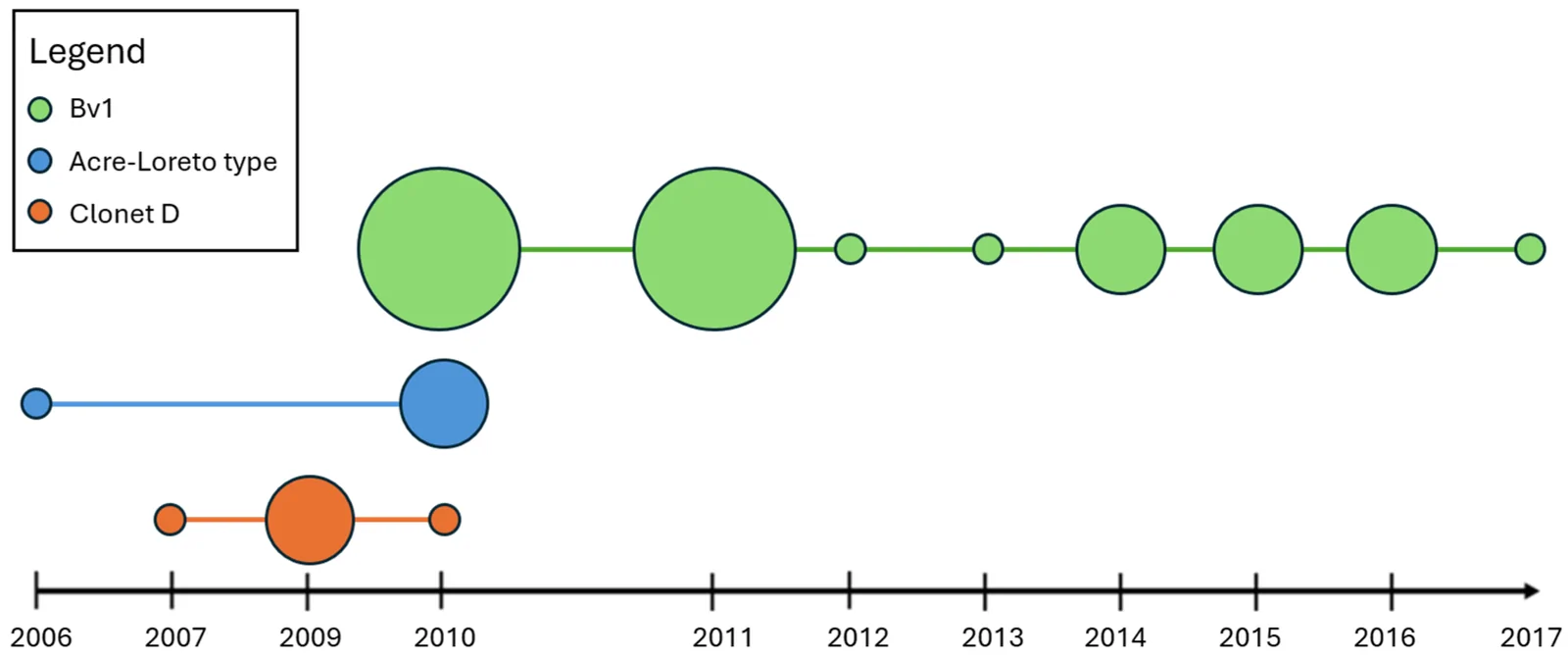
Circulating *P. falciparum* populations clonal lineages in Loreto region. The figure shows a transition in circulating lineages from 2006 to 2017. This scenario is characterized by the sole presence of the Bv1 type lineage since 2011. Adapted from [46].

In the case of *P. falciparum*, NAMRU SOUTH conducted a pilot genomics study using historical samples collected between 2006 and 2017 from the Peruvian Amazon (Loreto) and the North Coast (Tumbes). The study found evidence of a clonal replacement in the Peruvian Amazon in 2011, likely driven by the Project for Malaria Control in Andean Border Areas (PAMAFRO) conducted from 2006 to 2010 [46]. Starting in 2011, all specimens sequenced in the Loreto region corresponded to the Bv1-type lineage (Figure 4). This clonal replacement was further supported by a larger study that showed that over 95% of all *P. falciparum* specimens collected in Loreto belonged to this lineage [47].

The Bv1-type lineage presents several drug-resistance-associated polymorphisms, conferring high-level resistance to chloroquine (CQ) and sulfadoxine-pyrimethamine (SP). This profile might have provided an indirect selective advantage in response to chloroquine treatment for *P. vivax* that is administered in the region. Furthermore, this lineage has a history of contributing to two major outbreaks in Peru [48,49] and was also reported in Colombia [50]. This turnover has important public health implications, as newly dominant clones can affect clinical presentation, transmission rates, drive outbreaks, or change drug sensitivity profiles, requiring changes in existing control strategies.

NAMRU SOUTH has additionally partnered with other institutions working in Peru to test highly multiplexed deep sequencing assays, such as the *Pf* and *Pv* AmpliSeq panels or molecular inversion probes (MIPs) [51,52,53,54]. These panels allowed for high-throughput genotyping of drug resistance and population genetic markers, providing a highly accurate and cost-effective alternative to WGS that can be employed directly by National Malaria Control Programs. From a public health policy perspective, our genomic surveillance utilizing these panels found no evidence of artemisinin resistance mutations in circulating *P. falciparum* lineages in Peru. This finding is critical for local health authorities, providing the evidence base required to support the continued use of artemisinin-based therapies as the first-line treatment in the region.

### 6.4. Case Study 4: Use of Genomics for Tracking Antimicrobial Resistance

NGS methods offer an expanded toolkit for understanding different aspects of antimicrobial resistance (AMR) in bacterial species from the ESKAPE group (*Enterococcus faecium*, *Staphylococcus aureus*, *Klebsiella pneumoniae*, *Acinetobacter baumannii*, *Pseudomonas aeruginosa*, and *Enterobacter* spp.), which are pathogens associated with healthcare-associated infections that frequently exhibit resistance to multiple antibiotic classes, as well as in sexually transmitted infection (STI) bacteria such as *Neisseria gonorrhoeae*. Information obtained from sequencing includes detecting horizontally transferable AMR genes, virulence genes, high-risk clones, and identifying closely related strains that may be circulating and disseminating in a specific area [55]. In addition, integrating genomic tools into surveillance allows for not only the tracking of AMR gene trends, which can provide critical information on changes in incidence, but also provides additional insights into outbreaks in hospital settings, thus allowing for the implementation of strategies to decrease costs and reduce mortality [56]. Furthermore, it can be used for environmental monitoring to detect resistance reservoirs through metagenomic analysis [57].

As part of NAMRU SOUTH’s research on AMR in Peru, there have been reports of different ESKAPE pathogens carrying genes that produce carbapenemases, enzymes that confer resistance to most beta-lactam antibiotics, including carbapenems. One report identified the VIM-2 carbapenemase in *Pseudomonas aeruginosa* at a military hospital, while another reported NDM-1 in *Acinetobacter baumannii* detected in the Peruvian Amazon region [58,59]. These reports build upon findings from various studies in Peru that have utilized sequencing methodologies to describe circulating AMR genes and sequence types (STs). For instance, researchers described a highly clonal lineage of carbapenem-resistant *Acinetobacter baumannii* belonging to ST-2 that carried *bla*_OXA-23-like_ or *bla*_OXA-24-like_ genes in a Peruvian hospital [60]. Additionally, the pandemic ST-147 clone of *Klebsiella pneumoniae*, harboring multiple AMR genes, was recently identified in southern Peru [61]. In an extended context, various clones of ESKAPE pathogens harboring AMR genes have been detected across Latin America, showing an increasing trend over the past few years [62]. For example, there are reports of ST-309 *Pseudomonas aeruginosa* carrying the carbapenemase gene *bla*_VIM-2_ isolated from a clinical specimen in Brazil [63]. In the same country, a case of methicillin-resistant *Staphylococcus aureus* ST-88 was reported, which presented genes associated with resistance to beta-lactams and macrolides [64].

In addition to the ESKAPE group, NAMRU SOUTH has isolated *Neisseria gonorrhoeae* from clinical specimens, some of which were characterized by WGS as part of global gonococcal surveillance. Among the most significant findings, the isolates from Peru were grouped into five distinct clusters, including two isolates with reduced susceptibility to azithromycin that harbored chromosomal resistance determinants [65]. Furthermore, the identification of these AMR genes allows for the implementation of targeted interventions to prevent the spread of highly resistant organisms.

## 7. NGS Applications for Infectious Diseases Control and Prevention

Influenza viruses are one of the best examples of how genomic surveillance can guide public health interventions, as their rapid evolution through antigenic drift requires frequent updates of seasonal vaccine formulations [66,67]. WGS allows for high-resolution characterization of circulating viruses, facilitating the identification of emerging influenza virus genetic clades, reassortment events, mutations associated with antigenic changes, and markers of antiviral resistance, as discovered by NAMRU SOUTH during the monitoring of avian influenza in wild birds in Peru [18]. These genomic data complement traditional antigenic characterization and epidemiologic surveillance used by the Global Influenza Surveillance and Response System (GISRS) to formulate recommendations for Northern and Southern Hemisphere influenza vaccines [68].

Regional respiratory surveillance and sequencing efforts conducted by NAMRU SOUTH across Latin America have generated influenza virus genomes from countries with diverse ecological and epidemiological settings, improving representation of a region that has historically been under-sampled in global datasets [18,69,70,71]. Timely submission of influenza sequences to the Global Initiative on Sharing All Influenza Data (GISAID), together with data sharing with the Department of War’s (DoW) Global Emerging Infections Surveillance (GEIS) program [72] and U.S. Air Force School of Aerospace Medicine (USAFSAM) in addition to other host country surveillance partners, strengthens situational awareness of viral evolution across the Americas and supports comparative phylogenetic analyses with globally circulating strains [73] which are annually presented at the influenza Vaccines and Related Biological Products Advisory Committee (VRBPAC) meetings [74].

Although vaccine strain selection relies on the integration of virologic, antigenic, serologic, and epidemiologic evidence, regional genomic data provide critical information for identifying viruses that may exhibit antigenic divergence from current vaccine components and for prioritizing isolates for further phenotypic characterization [73,74]. Consequently, NAMRU SOUTH sustained respiratory pathogen genomic monitoring (e.g., influenza virus, SARS-CoV-2) in Latin America enhances regional preparedness, contributes to global respiratory pathogen risk assessment, and provides essential evidence supporting vaccine strain evaluation for both Northern and Southern Hemisphere influenza seasons.

## 8. Future Directions

Low- and middle-income countries often bear a disproportionate burden when it comes to global challenges, particularly in areas like life science research and infectious disease surveillance, which are essential for effective control and prevention measures. The rapid advancement of genomics and pathogen surveillance has highlighted a significant gap between the technologies available for data generation and the capacity to analyze, store, and effectively utilize that genetic data. To build long-term bioinformatics capability, establish sustainable data infrastructure, and foster strong regional partnerships, it is crucial to develop global public health frameworks. These frameworks should aim to transition from short-term, donor-dependent projects to resilient national health systems. In this context, sustainable financing models are essential for supporting data resources and related services within a long-term data infrastructure. This is particularly important because bioinformatics capacity, including wet-lab components, can only be maintained when it is integrated into active, well-funded, and locally led research programs [75].

To ensure accurate interpretation of complex raw data, state-of-the-art sequencing equipment must be paired with a skilled workforce. Along with sustainable funding, focused training methods, such as the “Training-of-Trainers” (ToT) approach, should be developed to facilitate efficient knowledge transfer that can be retained by the selected cohort. Collaborative initiatives, like those led by the Africa CDC and other regional institutions, exemplify successful ToT model implementation. These initiatives demonstrate strong collaboration between high-income and low-income countries [75,76]. By training a selected group of professionals within National Public Health Laboratories, countries can establish self-sustaining educational hubs that cascade knowledge to junior staff, creating a powerful multiplier effect. The integration of bioinformatics capabilities into public health surveillance should focus on applied genomic epidemiology relevant to regional needs. This approach equips analysts with practical workflows for real-time outbreak response, tracking AMR, and conducting pathogen surveillance.

Furthermore, Artificial Intelligence (AI) platforms are poised to democratize bioinformatics and data analytics by lowering computational barriers and optimizing analytical workflows. At NAMRU SOUTH, a Department of War laboratory, AI applications are strictly regulated to maintain compliance and robust data security. Current implementations focus on accelerating pipeline development, refining the codebase, integrating modular bioinformatic components, and troubleshooting technical bottlenecks. While future AI advancements will undoubtedly maximize workflow efficiencies and expand accessibility for non-experts, human oversight remains paramount. Rigorous curation by subject matter experts is essential to validate automated outputs, correct AI-introduced errors, and guarantee the reproducibility and scientific integrity of resulting datasets.

Pathogens do not respect geopolitical borders, making isolated national efforts inadequate for managing global health threats. This underscores the importance of strong regional partnerships. Establishing well-equipped “Centers of Excellence,” similar to those in Africa, develops regional sequencing hubs. These hubs allow resource-rich countries to act as technical anchors, providing support to neighboring under-resourced nations through sample sharing, technical mentorship, and access to high-throughput sequencing.

In summary, organizations such as NAMRU SOUTH and other established key players in the region possess the capabilities to support genomic surveillance activities, which are critical for controlling and preventing infectious diseases, particularly along cross-border transit corridors. By addressing infectious disease threats at their source, NAMRU SOUTH and its partners can provide data for Force Health Protection, while simultaneously strengthening partner nations’ public health infrastructure through Global Health Engagement. Enhanced collaboration within regional networks can harmonize surveillance efforts and align laboratory standards, enabling public health authorities to track disease transmission dynamics in real-time. Finally, countries can develop self-reliant public health ecosystems by integrating bioinformatics capabilities, infrastructure development, and regional network collaboration into a single cohesive strategy. This approach ensures that regional health challenges are addressed with locally driven and data-informed solutions.

## Figures and Tables

**Table 1:** Historical summary of genomic and partial-genomic sequences generated and deposited into public repositories through NAMRU SOUTH surveillance activities.

Organism/Pathogen	Years	Sequences Publicly Shared
Dengue virus (DENV-1–4)	2000–2015	155
Oropouche virus (OROV)	2022–2024	9
Ilheus virus (ILHV)	2025	1
Echarate virus variant	2019	1
Saffold virus (SAFV)	2012	1
SARS-CoV-2	2021–2026	729
Influenza virus (A and B)	2022–2026	685
*Plasmodium falciparum*	2003–2025	431
*Plasmodium vivax*	2011–2025	1327

**Table 2:** Identification of pathogen and vector species by ONT DNA metagenomic sequencing of ticks and fleas from dogs in Peru.

Species	State	*Rickettsia* qPCR Ct	Filtered Reads/Mean Length (bp)/Mean Quality Score (Q)	Pathogen Identification by Epi2Me (Reads)	Pathogen Identification by Minimap2 and BLASTN (Reads)	Vector Identification (COI) by Minimap2/BLASTN (Reads, Highest % Identity)
*Rh. sanguineus*	Amazonas	N.D.	472,330/1065.3/12.3	-	-	*Rh. sanguineus* (158, 97)
*Rh. sanguineus*	Amazonas	31.27	313,445/786.2/12.3	-	-	*Rh. sanguineus* (142, 97)
*Rh. sanguineus*	Amazonas	N.D.	1,208,810/619.1/12.3	-	-	*Rh. sanguineus* (118, 98)
*Rh. sanguineus*	Amazonas	N.D.	1,550,858/730.5/12.3	-	-	*Rh. sanguineus* (56, 98)
*Rh. sanguineus*	Amazonas	33.69	2,096,549/628.8/12.4	-	-	*Rh. sanguineus* (238, 96)
*Rh. sanguineus*	Cajamarca	N.D.	675,062/1004.7/12.3	-	-	*Rh. sanguineus* (127, 98)
*Rh. sanguineus*	Cusco	N.D.	1,528,903/813.2/12.3	-	-	*Rh. sanguineus* (104, 97)
*Rh. sanguineus*	Loreto	36.03	545,099/1028.8/12.4	*O. tsutsugamushi* (18)	-	*Rh. sanguineus* (283, 97)
*Ct. canis*	Loreto	37.19	18,999/737.7/12.6	*R. asembonensis* (290)	*R. felis*-like (6)	*Ct. felis* (1, 95)
*Ct. felis*	Amazonas	22.62	141,588/1046.6/13	*R. asembonensis* (606)	*R. felis*-like (20)	*Ct. felis* (46, 97)

Note: No detection (N.D.).

## Data Availability

The original contributions presented in this study are included in the article. Further inquiries can be directed at the corresponding author.

## Abbreviations

The following abbreviations are used in this manuscript:

AMR	Antimicrobial Resistance
BDRD	Biological Defense Research Directorate
CDC	Centers for Disease Control and Prevention
DENV	Dengue Virus
DoW	Department of War
GEIS	Global Emerging Infections Surveillance
GISAID	Global Initiative on Sharing All Influenza Data
GISRS	Global Influenza Surveillance and Response System
MMS Americas	Malaria Molecular Surveillance network
NAMRU SOUTH	U.S. Naval Medical Research Unit SOUTH
NGS	Next-Generation Sequencing
ONT	Oxford Nanopore Technologies
OROV	Oropouche Virus
PCR	Polymerase Chain Reaction
SISPA	Sequence-Independent, Single Primer Amplification
SOUTHCOM	U.S. Southern Command
ST	Sequence Type
STI	Sexually Transmitted Infection
SWGA	Selective Whole-Genome Amplification
ToT	Training-of-Trainers
USAFSAM	U.S. Air Force School of Aerospace Medicine
VBD	Vector-Borne Disease
VRBPAC	Vaccines and Related Biological Products Advisory Committee
WGS	Whole Genome Sequencing
